# Data-Driven Approach for Upper Limb Fatigue Estimation Based on Wearable Sensors

**DOI:** 10.3390/s23229291

**Published:** 2023-11-20

**Authors:** Sophia Otálora, Marcelo E. V. Segatto, Maxwell E. Monteiro, Marcela Múnera, Camilo A. R. Díaz, Carlos A. Cifuentes

**Affiliations:** 1 Telecommunications Laboratory (LabTel), Electrical Engineering Department, Federal University of Espírito Santo (UFES), Vitória 290075-910, Brazil; sophia.gonzalez@edu.ufes.br (S.O.); marcelo.segatto@ufes.br (M.E.V.S.); camilo.diaz@ufes.br (C.A.R.D.); 2 Federal Institute of Espírito Santo (IFES), Serra 29040-780, Brazil; maxmonte@ifes.edu.br; 3 Bristol Robotics Laboratory, University of the West of England, Bristol BS16 1QY, UK; marcela.munera@uwe.ac.uk

**Keywords:** muscle fatigue, electromyography, inertial sensors, Optical Fiber Sensors, machine learning

## Abstract

Muscle fatigue is defined as a reduced ability to maintain maximal strength during voluntary contraction. It is associated with musculoskeletal disorders that affect workers performing repetitive activities, affecting their performance and well-being. Although electromyography remains the gold standard for measuring muscle fatigue, its limitations in long-term work motivate the use of wearable devices. This article proposes a computational model for estimating muscle fatigue using wearable and non-invasive devices, such as Optical Fiber Sensors (OFSs) and Inertial Measurement Units (IMUs) along the subjective Borg scale. Electromyography (EMG) sensors are used to observe their importance in estimating muscle fatigue and comparing performance in different sensor combinations. This study involves 30 subjects performing a repetitive lifting activity with their dominant arm until reaching muscle fatigue. Muscle activity, elbow angles, and angular and linear velocities, among others, are measured to extract multiple features. Different machine learning algorithms obtain a model that estimates three fatigue states (low, moderate and high). Results showed that between the machine learning classifiers, the LightGBM presented an accuracy of 96.2% in the classification task using all of the sensors with 33 features and 95.4% using only OFS and IMU sensors with 13 features. This demonstrates that elbow angles, wrist velocities, acceleration variations, and compensatory neck movements are essential for estimating muscle fatigue. In conclusion, the resulting model can be used to estimate fatigue during heavy lifting in work environments, having the potential to monitor and prevent muscle fatigue during long working shifts.

## 1. Introduction

Muscle fatigue (MF) is defined as the inability to sustain a predictable maximal force during voluntary contraction [1] and the reduction of the capacity to generate force or power output [2]. MF can be associated with musculoskeletal disorders (MSDs) affecting workers’ ability to perform repetitive activities over long periods [3]. MSDs are one of the major health problems related to physical labour [4]. These can negatively affect people’s quality of life by being unable to perform daily living activities, self-care and work. Therefore, the ability to work is influenced by psychological, cognitive and social factors and the physical pain caused by MSDs [3].

Estimating MF is relevant for applications in sports, medicine, and ergonomics [5]. In athletes, MF occurs due to high-intensity training, leading to musculoskeletal injuries and reduced motor performance [6,7]. In medicine, the MF analysis in the diagnosis of neuromuscular diseases is essential [8]. Also, ergonomics has procedures for reducing local muscular workloads for employers, occupational health-related staff, and workers [9]. Manual lifting is commonly used in work environments to transfer or carry objects [10]. The improper lifting and long-term activities can contribute to excessive MF leading to occupational injuries, and affecting workers’ productivity, safety, and well-being [11]. The biceps brachii is the primary muscle performing manual handling and repetitive lifting tasks [12].

Different kinematic changes can occur during the fatigue state, such as decreased motor performance, speed, and Range of Motion (ROM) [13]. Also, MF alters the coordination of muscle activity, joint kinematics, and postural control, where it is observed that people are continuously changing their movements to maintain the performance of a task [14,15]. These changes are used to estimate the fatigue state of the person using different techniques, such as invasive and non-invasive techniques and subjective scales. Biologically, it can be estimated by blood samples or muscle biopsies [16]. However, these invasive methods estimate post-activity MF and do not generate real-time information.

On the one hand, non-invasive techniques exist to estimate and evaluate MF using surface electromyography that can be analyzed using amplitude, spectral, time-frequency, and nonlinear parameters [16]. For instance, Halim et al. studied muscle activity through surface electromyography (sEMG) sensors during manual lifting activities at different heights and loads. The results showed that the load mass and lifting weight significantly influence the mean power frequency of the two muscles responsible for this activity [17]. EMG is also considered the gold standard method to estimate MF since it directly assesses the bio-electrical muscle function [18]. However, EMG readings are inaccurate in long-term working environments due to skin sweating and electrode contact [19].

On the other hand, there are low-cost wearable devices such as Inertial Measurement Units (IMUs), goniometers and Optical Fiber Sensors (OFSs) for MF estimation. The IMUs can be used to evaluate and estimate MF by studying posture characteristics [20], and kinematic changes [21]. Mamam et al. collected data from four IMUs (located at the ankle, hip, wrist, and torso) and a heart rate sensor to detect fatigue in different industrial tasks. The results showed the identification of localized MF in the back with a single wearable sensor using seven characteristics [22]. Also, when running, changes in knee flexion angle have been evidenced using IMUs. Marotta et al. used six joint angles for feature extraction, among other biomechanical parameters, where joint angles resulted in higher fatigue-detection accuracy [23]. The goniometer has also been used as an indicator of localized muscle fatigue in static or dynamic tasks, where the drop in the joint angle to a set threshold indicates fatigue [24]. In addition, it can be used to find the angular displacements of the shoulder joint in conjunction with accelerometers, which is a determinant of fatigue [25]. Finally, it has been used to assess muscle fatigue to establish the boundaries of fatigue states in EMG signals, using indicators of elbow angle and oscillation, i.e., standard deviation [26].

Additionally, the OFSs present multiple advantages over a goniometer related to its immunity to electromagnetic interference, flexible structure, lightweight, and robustness [27]. The principle is based on intensity variation due to its simplicity and cost-effectiveness, where displacements and disturbances change the light intensity to obtain variables such as curvature, temperature, and pressure [28]. OFSs are currently used as angle sensors for accurately estimating the joints’ angles [29,30]. Also, optical fiber angle sensors are suitable due to their higher resistance to impact and vibrations [31]. Concerning fatigue, OFSs can be used to assess joint angle alterations. Yang et al. confirmed that after performing a repetitive pointing task, shoulder fatigue caused angular changes in the trunk, shoulder and elbow [32]. However, no report in the literature links IMUs and Optical Fiber sensors for muscle fatigue estimation, considering their advantages.

To correlate the sensor measurements, subjective scales are used and queried in real time to associate the acquired data with an individual’s perceived exertion. For instance, the Borg scale is a method of rating perceived exertion on a 0–10 scale and has previously been related to objective measures such as EMG [33]. This questionnaire is commonly used to monitor the feedback of physiological, psychological and situational factors to evaluate how easy or difficult a task is and the level of tiredness [34]. It has been used to monitor fatigue in manual material handling tasks, treadmill running, squatting, and simulated construction activity [35].

Due to the disadvantages of EMG sensors in estimating fatigue in work environments for extensive periods, this article aims to explore an alternative for MF estimation using wearable and non-invasive sensors such as OFS and IMU sensors and a subjective measurement using the BORG scale. Considering that EMG sensors are commonly used for fatigue estimation, it is expected to observe the importance of this sensor in estimating muscle fatigue and its contribution to other sensors. EMG sensors can evaluate and validate the MF of the biceps brachii, OFS can monitor the elbow joint angle, and IMU sensors can provide additional information about the movement of the arm and compensatory strategies in the trunk during induced fatigue. The validation will be performed using different machine learning methods using the three sensors to train the models and then determine the most suitable method for predicting MF of the biceps brachii. This will explore alternatives for estimating and monitoring work fatigue in long working shifts of heavy lifting due to EMG’s disadvantages with long-term use.

## 2. Materials and Methods

This study uses a system of three sensors that measure muscle fatigue through physiological and kinematic parameters in a repetitive elbow activity during heavy lifting. To perform this local fatigue analysis, an essential muscle in heavy lifting is used, which is the biceps brachii [12]. The biceps curl activity is selected since it has also been used to analyze and monitor biceps brachii muscle fatigue [36,37,38].

### 2.1. Materials

#### 2.1.1. Optical Fiber Sensor

**Figure 1:**
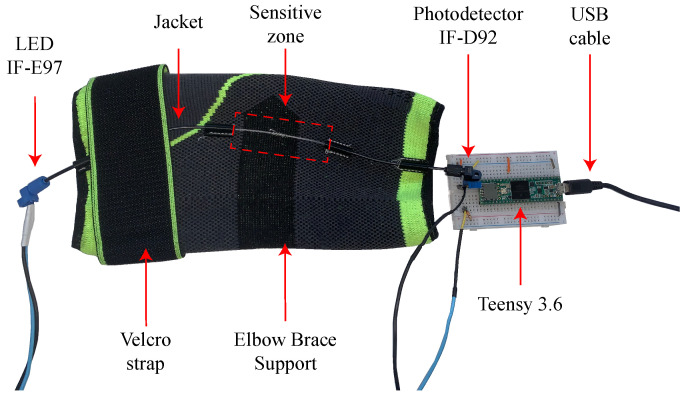
Elbow brace support used to attach the OFS and the electronic components.

The OFS was threaded through an elbow brace support made of a high-elastic synthetic fabric (Elastane), fastened with velcro. The polymer optical fiber (SH4001, Mitsubishi Chemical Co., Charlotte, NC, USA) estimates the elbow’s angle. To detect voltage changes, a Light-Emitting Diode (LED) IF-E97 and a phototransistor IF-D92 (Industrial Fiber Optics, Tempe, Arizona, USA) are placed on opposing sides of the fiber. A sensitive zone enhances the sensor response; this increases the optical power losses and generates greater voltage changes in response to the elbows’ flexion and extension movements [39]. Lastly, a microcontroller Teensy 3.6 (PJRC, Portland, OR, USA) with a 16-bit analogue-to-digital converter (ADC) was used to acquire the data from the OFS. The components are shown in Figure 1.

The characterization of the OFS is performed with all of the subjects performing two known angles of flexion (0°) and extension (140°) before the fatigue test for proper inter-subject characterization. These values were verified using a camera as a reference system to record the elbow movements and subsequently use the software Kinovea 0.9.4 to track the angles.

#### 2.1.2. Electromyographic Sensor

Subjects were instrumented with surface electrodes after sterilizing the skin surface using alcohol pads [40] and with an EMG acquisition module (Shimmer3 EMG Unit, Shimmer, Dublin, Ireland). The sensor was located in the biceps brachii of the subject’s right arm to register the muscle activity. For signal acquisition, a sampling frequency of 1024 Hz was used [41]. The instrumentation procedure and electrode placement followed the SENIAM guidelines [42]. Subsequently, the subject performs the maximum voluntary contraction (MVC) to normalize the intersubject measurements of the signals. They must execute a muscular contraction of the biceps brachii and maintain it for 5 s, followed by 10 s of relaxation. Finally, the MVC is averaged from 3 consecutive measurements. This muscle is evaluated for its relevance in manual handling and repetitive lifting tasks, essential for analyzing MF [12].

#### 2.1.3. Inertial Sensors

Two Shimmer3 IMUs (Shimmer3 IMU Unit, Shimmer, Ireland) were located on the subject’s anterior carpal region (wrist) of the right arm and in the neck at the level of the C7 vertebra of the spine. Before the start of the session, data were acquired with the person in an anatomical position for 1 min to calibrate all the sensors. The data were acquired at a sampling frequency of 128 Hz [43]. The IMU at the wrist is essential to measure upper limb-related activities [44]. The IMU in the column can observe how fatigue due to repetitive upper limb tasks can affect neck compensation movements [45].

#### 2.1.4. Borg Scale CR10

**Figure 2:**
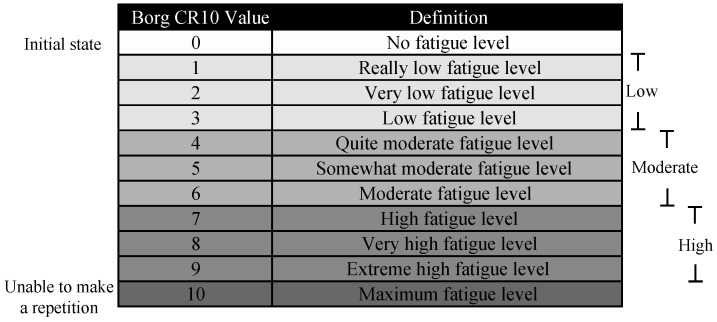
Borg CR10 Scale.

To identify the different levels of fatigue subjectively, the Borg scale was used to evaluate the rate of perceived exertion (RPE) [46]. The Borg scale is commonly used to assess the local MF in the biceps brachii and the effect of MF on wrist joint position [47,48]. This is shown in Figure 2, where the three fatigue states are established as Low Fatigue (LF), Moderate Fatigue (MOF), and High Fatigue (HF). In the initial state, all participants were in state 0 according to the multidimensional fatigue inventory, i.e., in a fatigue-free condition [49] (see Appendix A). In addition, when the participant reached a scale of 10, the test was concluded since they could no longer generate another repetition. Participants were introduced to the scale before the trials with verbal and visual explanations. This scale was asked every 20 s to all participants during the fatigue test.

### 2.2. Subjects

This study involved the voluntary participation of 30 subjects, 14 female and 16 male, who performed a repetitive weight-lifting activity. Inclusion criteria included being healthy subjects between 18 and 30 years and also being in a non-fatigue state according to the Multidimensional Fatigue Inventory. The age range of healthy young people is chosen since the reliability of strength tests in older populations may be lower due to decreased muscle strength and joint stability [50]. To establish the user’s non-fatigued condition, this questionnaire assessed five types of fatigue, such as general fatigue, physical fatigue, mental fatigue, reduced motivation, and reduced activity. The exclusion criteria excluded subjects who have suffered an arm fracture, with musculoskeletal or systemic disorder and any known impairment of postural control or motor function. The experimental protocol and the purpose of the study were explained to all subjects, and informed consent was obtained before the study. The mean and standard deviation (mean ± std) of the demographic data of all subjects are shown in Table 1.

### 2.3. Experimental Protocol

This is a prospective observational study. Subjects are initially instrumented with EMG sensors in the biceps brachii and perform the MVC of the muscle. Subsequently, they are instrumented with the 2 IMUs sensors and the elbow brace support with the OFS, and a calibration test is performed where they remain 1 min in anatomical position. This allows a baseline measurement for each sensor and subtracts the offset for subsequent measurements.

**Figure 3:**
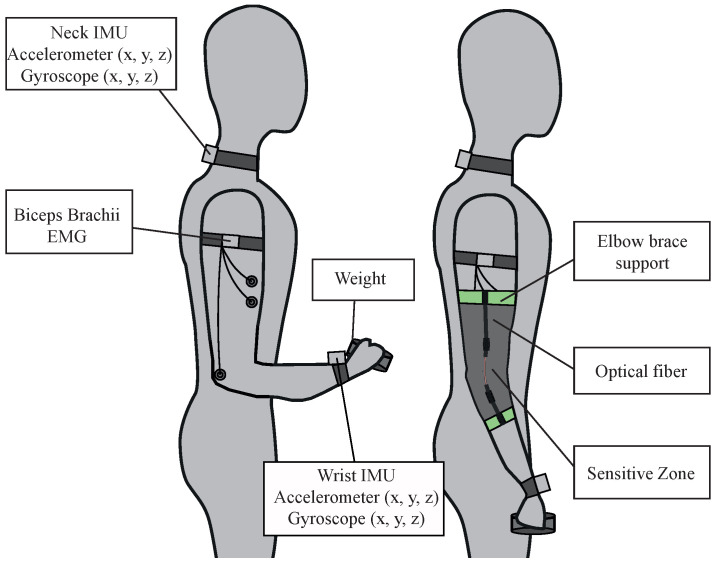
Experimental setup. Participants were instrumented with an EMG sensor and two inertial sensors as illustrated on the left and then instrumented with the optical fiber sensor on top as shown on the right.

Afterwards, a warm-up phase should be performed with a lower weight than the fatigue phase to prepare the participants’ muscles. The participants perform the biceps curl activity for ten repetitions using a load of 1 kg for women and 2 kg for men [51]. Figure 3 illustrates the placement of the IMUs, EMG, OFS, and weight.

Finally, subjects perform the biceps curl activity with a higher load (2.5 kg for women, 4.5 kg for men) [52] until reaching MF, i.e., reaching ten on the Borg scale or failing to perform the full ROM. Results have been previously demonstrated in the biceps muscle that increasing the load generates greater MF in repetitive lifting activities in this muscle [53]. Then, the sensors were removed from the person, and they were instructed to perform three minutes of arm muscle stretching to avoid future local pain.

### 2.4. Procedure of Proposed Fatigue Classifier

**Figure 4:**
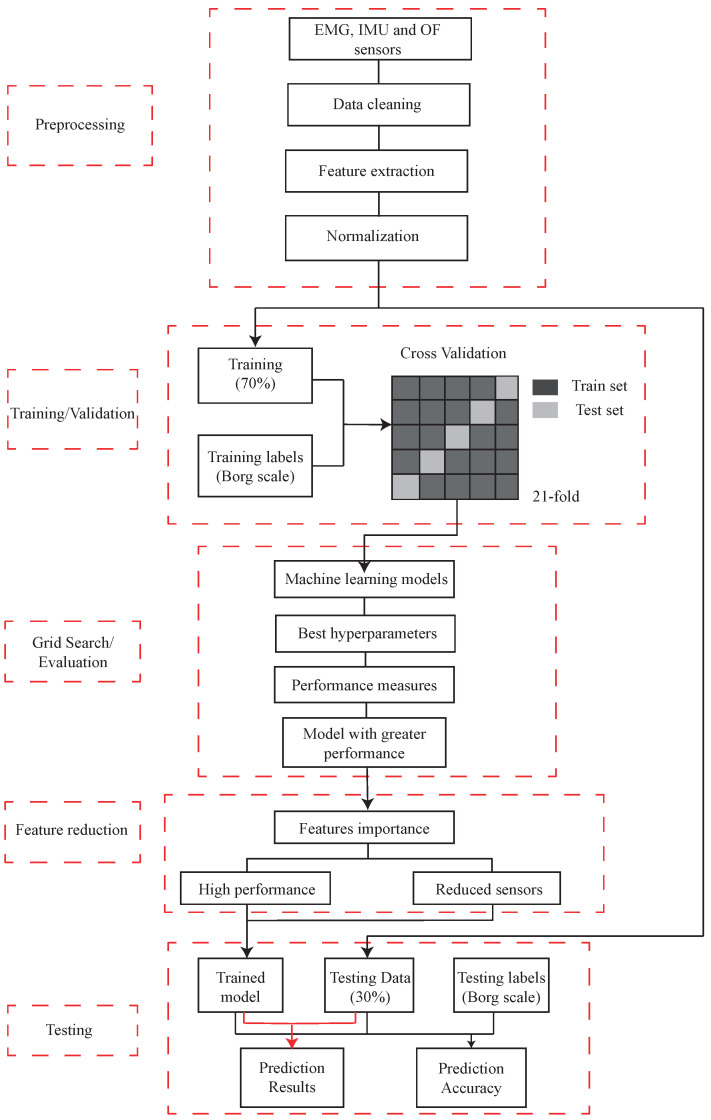
Flowchart showing an overview of the proposed procedure.

Figure 4 represents the MF detection algorithm process, divided into five stages: (1) processing data extracted from the sensors and feature extraction. Also, all of the features were normalized regarding the initial value corresponding to the average of the first window values each window represents a biceps curl repetition cycle) of each characteristic except for the frequency-related features; the next stage is (2) training and validation, where the data is separated into 70% for training where cross-validation is performed with a k = 21, and 30% for the subsequent test. The training and testing labels correspond to the Borg scale values. The machine learning models are applied, and a (3) grid search is performed to evaluate the best hyperparameters of each model and thus choose the model with the best performance; following this, a (4) feature extraction analysis is performed according to the best performances and the number of sensors used; finally, (5) the data is tested with the model and the evaluation metrics of the machine learning model are obtained.

#### 2.4.1. Data Processing

The processing begins by interpolating the values of the Borg scale and assigning each value to the corresponding time to consider the duration of the fatigue states. This interpolation is performed to obtain a greater number of values and to divide the data from all the sensors. All sensor signals are divided according to the three fatigue states in Figure 2.

**Figure 5:**
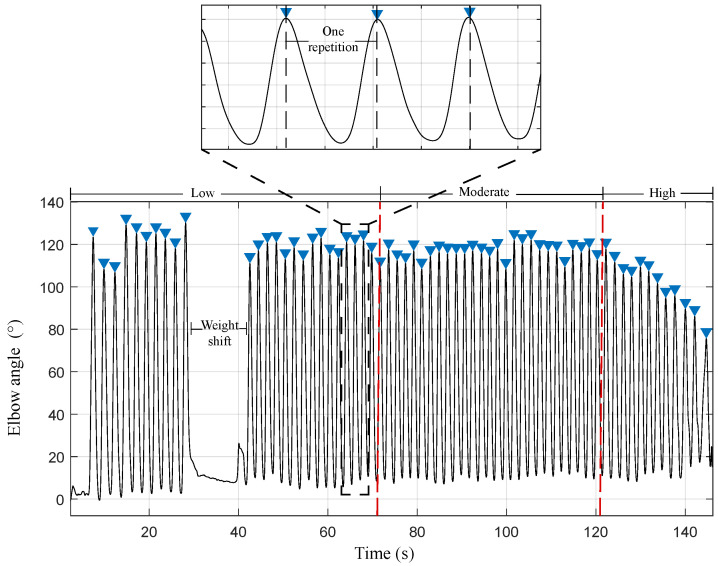
Optical fiber sensor signal. The complete repetition of the biceps curl (window) represents a peak-to-peak distance. The red lines indicate the separation of the fatigue states.

For the OFS data, a low-pass filter with a cut-off frequency of 0.5 Hz was applied to eliminate and reduce noise. This value was found by using the Fourier transform of the signal. After this, the signal is divided into the three fatigue states, and the repetition cycles of the elbow are detected, being a cycle as the peak-to-peak distance, as shown in Figure 5. Therefore, the window length is defined as the biceps repetition cycle with a fixed size and without overlapping. From these windows, inter-subject features were extracted.

From each cycle of the OFS signal, eight characteristics are extracted: The normalized duration of each cycle, mean, standard deviation, Root Mean Square (RMS) value, ROM, mean frequency (MNF), median frequency (MDF), and instantaneous frequency (IMNF) of the signal. The cycle times extracted from this sensor were taken as a reference to extract the characteristics of the other sensors.

Concerning the EMG signal, a band-pass filter (4th-order Butterworth filter with cut-off frequencies of 15 Hz and 450 Hz) is applied to remove the noise and eliminate the baseline drift caused by motion and DC offset [54,55,56]. The signal is then normalized according to the MVC, rectified, and a 200 ms moving window filter is applied to obtain the signal envelope. Afterwards, the signal is divided into the fatigue states and repetition cycles for extracting seven features per cycle, including mean, standard deviation, RMS value, amplitude, MNF, MDF and IMNF.

Lastly, a moving average filter of 30 ms is applied to the IMU sensors (Gyroscope and Accelerometer in x, y, z) to reduce the noise. They are separated according to the fatigue states, and 4 characteristics per cycle are extracted, including mean, standard deviation, RMS value and signal amplitude. Time-amplitude-related parameters are sensitive to intrinsic and extrinsic factors and require normalization of the data to be able to compare inter-subject data. This estimation is not required for frequency-related characteristics [57,58]. Equation (1) was used for feature normalization. This was calculated regarding the initial value of each characteristic [59]. This is possible since, in the multidimensional fatigue questionnaire, the subjects began with a 0 or relaxed state.

(1) $$f_{n}=\frac{f_{i}}{f_{0}},$$

where $f_{n}$ is the normalized feature given by $f_{i}$, which corresponds to each feature extracted from the time window divided by $f_{0}$, which is the first feature extracted from the time window.

Based on the importance of measuring joint kinematics such as angle when performing fatigue exercises, the eight representative features of the joint position signal are calculated. During repetitive movements, an increase in movement variability is generated as fatigue develops [60]. Therefore, the mean value, the variability of movement (standard deviation), range of motion (ROM), movement duration (t_norm) and the frequency-related variables such as MDF, MNF and IMNF are calculated. In addition, IMU sensors are used because they are wearable and non-invasive sensors and are used to detect MF to measure linear and angular velocities [61,62]. For this reason, components related to the signals, such as their mean, std, RMS value and amplitude, are extracted. Finally, a widely used method for fatigue detection is EMG, where it has been studied that alterations and increases in muscle activity are generated during maximal contractions. It has been confirmed that for acceleration and EMG data, the average and RMS values are among the best parameters for feature selection [63]. For this reason, the signal is studied in amplitude (mean, std, RMS, and amplitude) and frequency (MDF, MNF, and IMNF) [64]. The IMNF value has been used to assess fatigue in elbow motion derived from the continuous wavelet transform (CWT), where the value decreased significantly between the non-fatigued and fatigued conditions [65].

Table 2 shows the 63 features used in the model, including the three sensors used in the study.

#### 2.4.2. Training and Validation

To reduce or eliminate bias in training data in Machine Learning Models, dataset splitting is commonly used [71]. The dataset can be divided into different sets, and in this study, the ratio 70:30 train/test split was used. The model is developed using the training dataset, and its prediction ability is evaluated using the testing dataset [72]. The training set is then divided into multiple sets and it is trained using cross-validation [71]. This data resampling method is used to prevent overfitting and evaluate predictive models’ ability. It applies a learning function to multiple data subsets and then evaluates the resulting models on different subsets, i.e., test sets or validation that are not used during training. An estimation of the final model’s performance is the average of the model’s performance on each subset [73]. Leave-one-out cross-validation (LOOCV) was used, where the number of folds equals the number of instances or subjects in the training set. In this case, 70% is equivalent to 21 subjects in the training data [74,75].

#### 2.4.3. Evaluation

There are statistical models, single classifiers, and ensemble models to predict fatigue. However, by the study’s approach, which is data-driven and application-dependent, no specific method will be the most effective for the particular application [22].

During the initial analysis, several machine-learning methods were evaluated, including Light Gradient Boosting (LGBM), Extra Trees (ET), Random Forest (RF), Bagging (BC), Decision Tree (DT), K-Neighbors (kNN), Support Vector Machine (SVM), and Logistic Regression (LR). However, the latter three algorithms were dismissed due to their weak performance. These algorithms presented accuracy performances below 88% and F1-scores below 86%. The Python package “Lazy Predict” was used to generate multiple models to determine the dataset’s most optimal machine learning model.

Subsequently, an optimization of the hyperparameters of each model is performed using the grid search method where, starting from a subset of hyperparameters, a complete search is performed to obtain the optimal hyperparameters that generate a greater performance in the algorithm [76]. This was performed on the first five best-performing classification algorithms, i.e., LGBM, ET, RF, BC and DT.

#### 2.4.4. Feature Reduction

Once the model with greater performance is selected from the training stage, feature reduction is performed to reduce the number of redundant variables, and the computational cost [77]. Feature selection chooses a specific amount in a subset of features to minimize redundancy and maximize the relevance of the class labels in the classification, such as information gain, relief, and fisher score [78]. This method has been previously used in muscle fatigue classification studies to optimize the features [79,80]. For this reason, in the present study analysis, where it is desired to observe the main features that can detect MF in upper-limb activities, feature selection is used considering the best-performing features and depending on the number of sensors in practicality and user comfort for future studies.

#### 2.4.5. Model Evaluation (Testing)

The accuracy, precision, recall, and F1 score metrics are evaluated to observe the test algorithm performance. The Accuracy (Equation (2)) measures the ratio of correct predictions (TP) in all fatigue states over the total number of instances evaluated (*N*).

(2) $$Accuracy=\frac{TP}{N}$$

Precision (Equation (3)) is used to measure the positive patterns that are correctly predicted (TP) over the total predicted patterns in the positive class, which is divided by the False Positives (FP), which are the instances that were labelled as one fatigue class by the model but belonged to another, i.e., how much variability repeated predictions show compared the true values [81].

(3) $$Precision=\frac{TP}{TP+FP}$$

Recall (Equation (4)) is used to measure the fraction of positive patterns that are correctly classified as fatigue states, dividing the TP into False Negatives (FN), which are the instances classified as other fatigue groups and finally F1score (Equation (5)) represents the harmonic mean between the recall and precision values [82,83].

(4) $$Recall=\frac{TP}{TP+FN}$$

(5) $$F1_{micro}score=\frac{Precision*Recall}{Precision+Recall}*2$$

## 3. Results

In total, there are 1240 records from which 70%, i.e., 868 went through the validation and training stage of the model where the information corresponds to 21 subjects, and LOOCV is used where the validation k corresponds to the number of subjects, i.e., k = 21. The other 30% of the records are evaluated as part of the model test corresponding to 372 records. Table 3 shows the number of samples of the three fatigue states in the testing stage. Low fatigue has the highest number of samples with 37%, followed by high fatigue with 36% of samples and moderate fatigue with 28% of samples.

**Figure 6:**
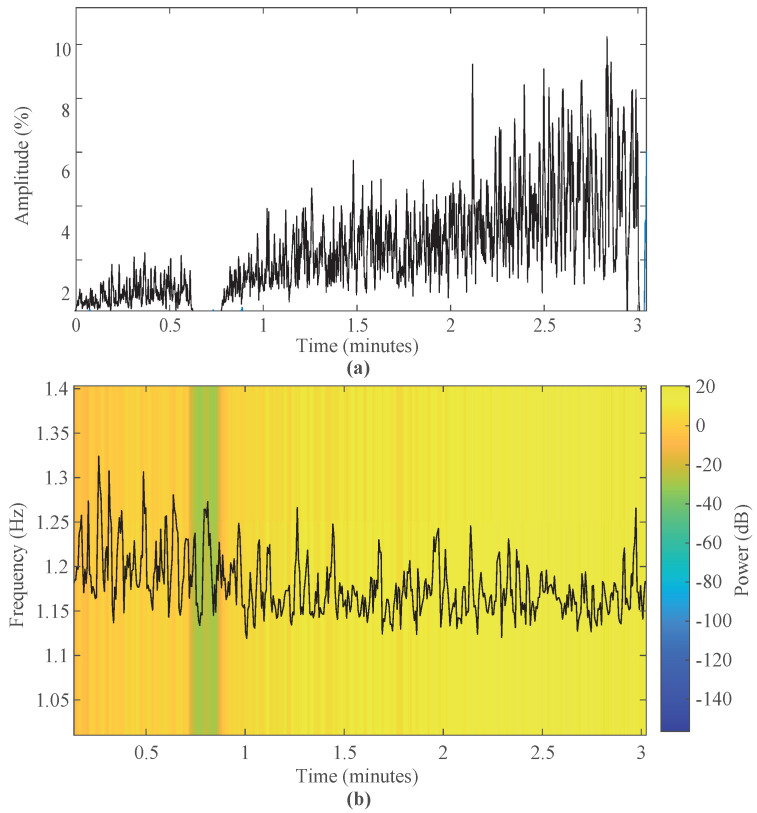
EMG of the biceps brachii in time and frequency of a test subject. (**a**) Amplitude muscle activity and (**b**) Instantaneous frequency throughout the test.

Figure 6a shows the increasing behaviour of the amplitude of the biceps brachii muscle activity in the fatigue test, and Figure 6b shows the decreasing behaviour of the IMNF during the test. In the section with no muscle activity and a green spectrum, the participants changed from a lower to a higher weight.

Table 4 presents the performance and evaluation metrics of the best five algorithms. Since the dataset is unbalanced, it is important to evaluate and compare models using precision, recall and F1-score [84]. To tune the hyperparameters, the grid search method was used to optimize the performance of the LGBM, RF, BC ET, and DT classifiers. This was performed using cross-validation, and the hyperparameters that resulted in the best-performing result were chosen for further analysis. This process was performed using Python libraries.

**Figure 7:**
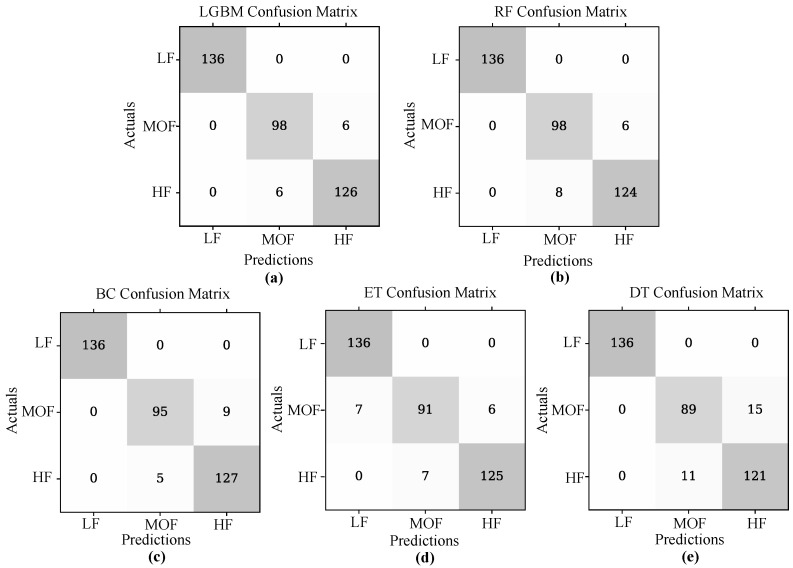
Confusion matrix of the best-performing machine learning models estimating muscle fatigue. Being (**a**) LGBM, (**b**) RF, (**c**) BC, (**d**) ET, and (**e**) DT classifiers.

The confusion matrix of the best models with parameter optimization is shown in Figure 7, where the values on the diagonal refer to the correctly estimated values of each fatigue state model (low, moderate, and high). The x-axis refers to the predicted class and the y-axis to the true class.

LGBM algorithm was tunned with the maximum number of leaves in each boosting round’s decision tree ’num_leaves’ set to 181, determining the balancing model complexity and generalization. Additionally, the fraction of data used in each boosting iteration ’bagging_fraction’ was set to 0.56, contributing to variance reduction. Finally, ’min_child_samples’ were set to 8, ensuring a minimum number of samples required to form a new leaf node.

The Random Forest (RF) classifier was tuned with ’n_estimators set in 1400 decision trees to collectively make predictions. The minimum number of samples required to split an internal node ’min_samples_split’ was set to 2, and the necessary minimum number to be at a leaf node ’min_samples_leaf’ was set to 1. To control the depth of each decision tree, ’max_depth’ was set to 40 and also managed model complexity. Lastly, bootstrapping ’bootstrap’, i.e., random sampling with replacement, is not used during tree building ’False’.

The Bagging Classifier (BC) was optimized with the maximum depth of the individual base estimator (decision trees) ’base_estimator_max_depth’ within the ensemble set at 20. Each base estimator considers only 70% (0.7) of the available features ’max_features’ when making split decisions. Lastly, the ’n_estimators’ parameter is set at 10, implying that the classifier employs ten base estimators in the ensemble. The Extra Trees Classifier (EC) was tuned with the criterion for splitting nodes set to ’log_loss’, indicating that the classifier employs logarithmic loss to make splitting decisions. The ’n_estimators’ parameter was set to 200, meaning the classifier uses 200 decision trees in its ensemble.

Finally, the Decision Tree (DT) classifier was tuned to enhance performance. The ’criterion’ was set to ’gini’, indicating that the Gini impurity is used as the criterion for making split decisions in the tree and ’min_samples_leaf’ was set to 1, implying that each leaf node must contain at least one sample. Also, the efficiency model of each classifier was extracted from the LazyClassifier, with times of 3.86 s for the LGBM, 0.96 s for the RF, 0.66 s for the BC, 0.45 for the Extra Trees and 0.08 s for the DT classifier.

**Figure 8:**
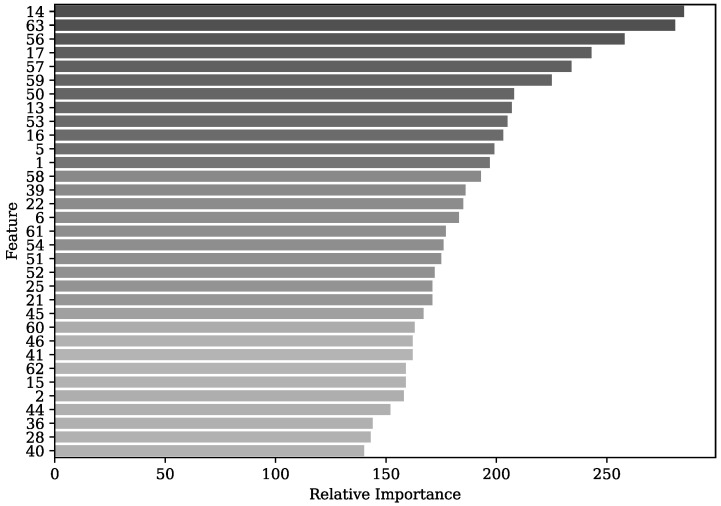
Feature importance ranking of the first 33 features with the LGBM classifier.

An analysis was performed on the feature importance of the best-performing model, i.e., the LGBM. The feature importance ranking is based on the split importance, which computes the number of times the feature is used in the LGBM classifier to represent the importance of that feature. This allows us to observe each feature’s contribution to improving the model’s predictive ability [85]. Figure 8 shows the feature importance ranking of the first 33 features out of 63 in total ordered from highest to lowest. It is observed that among the four most relevant characteristics for the model is the standard deviation of the acceleration in X of the IMU located in the wrist (14), the IMNF of EMG (63) and OFS (56) sensors, mean acceleration in Y of the IMU located in the wrist (17) and amplitude parameters such as the mean of the EMG signal (57).

According to the LGBM classifier, performance is explored with different features and varying the number of sensors to estimate MF. Considering the various combinations of sensors and each sensor separately, the results are presented in Table 5. It is observed that the best performance using the three sensors with the least number of features is obtained with 7 features with an accuracy and F1-score of 93.5%. When evaluating the sensors separately, EMG has the lowest performance with an accuracy of 78.8%, whereas the combination of IMUs obtained a more remarkable with ten features (92.2%) over each IMU separately, with 86.6% for the wrist IMU and 87.9% for the neck IMU. Finally, the combination of the two IMUs and, the OFS, obtained a higher performance of 95.4% in all metrics.

## 4. Discussion

This paper aims to explore an alternative for MF estimation using wearable sensors. Therefore, different combinations of EMG sensors, OFS and IMUs are discussed, including their advantages and limitations. The dataset tends to be unbalanced because, in fatigue studies, the amount of data acquired in low fatigue states tends to be greater than in fatigue states [35]. However, the highest difference in dataset size occurs between the low and moderate states with a difference of 8%, corresponding to a slightly unbalanced dataset. This implies that this percentage difference barely impacts the learner’s performance [86]. This was observed during the tests where the subjects remained in a low fatigue state over time, and the fatigue perception caused them to increase the scale to a high fatigue state rapidly. However, upon reaching this state, the muscles begin to condition due to the physical effort exerted, and most subjects remained in state nine before reaching MF. This is defined as long periods before failure where a burst of activity of the biceps muscle activity begins to appear due to more threshold motor units being recruited [87].

Obtaining physiological signals is an indicator to detect MF. EMG sensors provide information about muscle and physical fatigue by recording the electrical signal from the muscles. In the time domain, the fatigue is related to an increment of the EMG amplitude and in the frequency domain tends to decrease [88]. For this reason, MDF and MNF are extracted since data spectral analysis gives more information about the muscles’ function [89]. Due to a reduction in muscle fiber conduction velocity, the EMG power spectrum is shifted to lower frequencies during fatigue [90].

Figure 6a shows how the muscle activity increases, at the beginning, with a lower weight with values lower than 3% and increasing linearly with increasing weight, reaching higher values, reaching up to 8%. This result is also found in the study of the effects of MF in the biceps brachii, where the RMS increased as each contraction passed. Also, Figure 6b shows the frequency spectrum, where the IMNF gradually decreases as the subject experiences MF. This pattern is also observed in a study of elbow MF detection by continuous wavelet transform analysis where the IMNF decreases linearly between non-fatigue and MF [65].

Considering that multiple algorithms were evaluated, the five best-performing algorithms were LGBM, RF, BC, ET and DT. It can be observed in Figure 7 that the low fatigue state is correctly calculated in all algorithms and starts to mispredict moderate and high fatigue in the BC, ET and DT. These algorithms tend to predict moderate fatigue as high fatigue. This occurs due to the robustness of both algorithms. LGBM combines multiple sub-learners to create a strong learner to complete the learning task [91], and random forest uses random selection of a subset of features at each node, reducing the correlation between trees [92]. The Gradient Boosting Decision Tree (GBDT) is a commonly used machine learning algorithm for its efficiency, accuracy and interpretability. It has advantages in multi-class classification problems. Two techniques are integrated into the GBDT algorithm: gradient-based One-Side Sampling (GOSS) uses the gradients of instances and retains the larger gradients during downsampling to improve information gain estimation, and the second method, Exclusive Feature Bundling (EFB), which groups together regular important features, making the GBDT algorithm faster. These two methods create the LightGBM algorithm with improved performance on larger datasets as demonstrated in the current study [93].

In studies related to estimating MF in manual material handling using wearable sensors such as EMG, classifiers such as DT, SVM, kNN and RF were used to classify the risk, indicating DT as the algorithm that best identifies the risk using the NIOSH equation with a 99%. However, this study is limited since it was performed by only one subject, which is reflected in the high performances [94]. Similarly, in a study of upper limb MF detection, RF, SVM and LR were used, where the best performing algorithm was RF with an accuracy of 87.5% using EMG sensors [95]. As mentioned above, multiple studies focus on detecting MF using EMG sensors due to their high reliability. Therefore, IMNF, RMS, and mean EMG are among the most relevant characteristics in this study.

IMUs are widely used to estimate lower extremity fatigue in gait [20]. They have been used to distinguish gait patterns between fatigue and non-fatigue conditions using machine learning algorithms obtaining 96% with an SVM classifier [21]. In addition, they have been used to compare baseline and fatigue dynamic balance control which can capture motor disturbances [96]. Likewise, there are methods to detect fatigue in the upper-limb where a relationship has been found between the electrical activity and the angles of rotation of the forearm and upper arm, also presenting an increase in motion amplitude deviation of the upper arm [61]. In addition, it has been used to evaluate biceps fatigue using an IMU at the wrist, observing that fatigue reduces the angular velocity of the biceps, thus increasing the time to complete a set. By applying machine learning algorithms, an accuracy of 88% cross-subjects is observed using a feedforward neural network with 16 features [62]. Table 5 shows that only with seven features an accuracy of 93.5% is obtained and using only IMUs with ten features an accuracy of 92.2% is obtained.

Among the most relevant characteristics in IMU1 (wrist) are the standard deviation of the amplitude in x and the mean of the accelerometer signal in y, and in IMU2 (neck), the angular velocity in x and acceleration in x. It is relevant to emphasize that although IMU1 obtained more relevant characteristics because it is the IMU responsible for evaluating the flexion-extension movement, it is essential to complement it with the IMU2 as the person begins to perform compensatory movements that are reflected in the accelerations and angular velocities. Table 5 shows that with five features, IMU1 presents an accuracy of 86.6% and IMU2, with four features, presents a performance of 87.9%. However, by joining these inertial sensors, the performance increases to 92.2% with ten features. Fatigue-inducing repetitive movements alter both local features and reorganization of all movements. During a fatigued reaching activity, subjects elevated the shoulder, indicating a compensatory strategy to decrease the load on the shoulder muscle [97]. The videos showed that the subjects started performing lateral neck flexion and neck forward flexion as the movement and velocity decreased due to MF.

On the other hand, OFS have been used to measure elbow flexion, ensuring high sensitivity and repeatability. This type of sensor is used to monitor human joint angles in rehabilitation environments [98]. In addition, it has been shown to have similar results with potentiometer behaviour, presenting advantages over goniometers, IMUs and other types of OFS [99]. However, to the authors’ knowledge, it has not been used for fatigue detection by continuous assessment of elbow angle in a repetitive task. Considering that this sensor presents multiple advantages, highlighting its flexibility, lightweightness and immunity to electromagnetic interference, it can be used in work environments, monitoring the workers’ joint angles. Figure 8 shows that the most relevant features of the OFS are the IMNF, the standard deviation and the time to perform a repetition. As the subjects began to experience MF, they took longer to achieve the repetition until they could not perform the complete repetition. Therefore, they presented a reduced range of motion, increasing the standard deviation and decreasing the motion frequency.

The combination of these three sensors (EMG, IMU, OFS) according to Table 5 with the best classifier indicates a high performance with 33 features of 96.2% and with 7 features decreasing to 93.5%. Although EMG is a gold standard method to identify MF, these measurements tend to be misleading in prolonged working environments due to skin sweating and electrode contact. During hot and humid conditions in paper manufacturing and outdoor worksites, workers’ sweating increases, limiting the adhesion of the electrodes [19]. In contrast, wearable sensors such as IMUs and OFS offer robust solutions, providing accurate data in environments with high temperatures and humidity. The integration of these sensors obtained a 95.4% accuracy with 13 features being a highly reliable MF estimation. This leads to continuous monitoring throughout working days.

### 4.1. Practical Applications

The estimation of muscle fatigue employing wearable sensors holds significant potential in preventing MSD. By providing real-time biofeedback to users, these sensors can alert subjects when they start experiencing muscle fatigue. This information can be reported through an intuitive interface through visual or haptic feedback to adjust their posture or take preventive measures. Currently, this type of feedback is performed for general fatigue with drivers and operators, therefore, further research is needed for implementation with muscle fatigue [100].

Additionally, there are occupational exoskeletons designed to support workers in handling heavy loads during repetitive tasks [101]. These exoskeletons have control strategies that can generate appropriate reference signals to control the speed, torque, or impedance of the actuated limbs [102]. The algorithm can be implemented to improve the control strategies of the exoskeletons and generate greater human-robot interaction so that the device assists only when needed by the user.

### 4.2. Limitations and Future Works

Among the limitations of this study, since it is a preliminary analysis of the performance of wearable sensors and EMG sensors, there is the absence of the use of motion analysis cameras. It is a technique used in MF analysis and detection [19,103]. However, the present article focuses on the analysis of fatigue detection with wearable sensors to be used in future studies to monitor kinematic and kinetic variables in work environments. Nevertheless, in future works, when assessing workers dynamically, this system is expected to be included to evaluate compensatory movements in the labour environment.

Furthermore, the model’s efficiency is a limitation of the best-performing algorithms, with the LGBM having the longest execution time compared to the other algorithms. However, this is a factor that should be studied in real-time studies to see how it affects the worker’s biofeedback, and whether in this type of environment, an algorithm with shorter time and lower performance or with longer execution time and better performance is required.

Fatigue studies are commonly used for fatigue-inducing tasks. However, studies in real-life settings must be performed because although they simulate lifting a weight, it does not reflect the dynamic and complex working environment [104]. In addition, it has been proven that the subjects’ effort and perception are reduced in simulated tasks [35,105]; therefore, in this work, motivational words were used as encouragement for subjects to reach a true state of muscular fatigue where they could no longer perform an elbow flexion-extension repetition. For this reason, future work is expected to evaluate a work environment where the fatigue detection algorithm can be applied as a preventive measure in the work task that the users are performing.

## 5. Conclusions

MF is a physiological response due to prolonged physical exertion and can lead to musculoskeletal diseases. Therefore, detecting and monitoring fatigue is relevant, given the implications for workers’ well-being and safety. In this work, an algorithm for detecting biceps brachii MF was performed using wearable sensors such as IMUs, OFS and EMG sensors, obtaining an accuracy of 93.5% with seven features.

Using EMG analysis, the relationship of muscle activity with the progression of fatigue is confirmed in characteristics related to the IMNF and in amplitude to the mean and RMS values, observing its contribution in each state of fatigue. It is found that the behavior of the EMG signals tends to increase in the time domain and to shift to lower frequencies in the spectrum. This was reflected by obtaining the mean and the IMNF of the EMG among the best characteristics. However, the performance of exclusively this algorithm shows an accuracy of less than 80%.

When adding IMUs and OFS, the algorithm leads to higher performance when classifying the fatigue states, obtaining an accuracy of 95.4% with 13 features. This demonstrates the importance of evaluating elbow angle variations and their accelerations and angular velocities. This is demonstrated by the most relevant characteristics of the IMU1 (wrist) being the amplitude and standard deviation of the acceleration in vertical motion. However, the performance of this IMU1 is 86.6% and when combined with the IMU2 of the neck, an accuracy of 92.2% is obtained. This indicates the importance of monitoring the compensatory movements experienced by the subject during induced fatigue in these repetitive lifting activities. Similarly, with OFS, as they approached a fatigued state, the repetition time was longer, presenting a reduced ROM and decreasing the frequency of the flexion-extension movement.

Wearable sensors (IMUs and OFSs) perform better in real-world settings, where EMG sensors may be limited due to factors such as skin perspiration and electrode contact. This was observed with a performance of 95.4% with 13 features using IMUs and OFS compared to using the three sensors with more features (33) and a slight difference in performance with 96.2% accuracy. Future work is expected to focus on static and dynamic testing in a work environment using wearable sensors over a longer period during the workday to include feedback methods and alert users when experiencing muscle fatigue as a preventative measure.

## Figures and Tables

**Table 1:** Mean and standard deviation (mean ± std) of subjects’ demographic data.

Gender	Age (Years)	Weight (kg)	Height (cm)
Female	24.7 ± 3.4	61.1 ± 15.4	163.4 ± 7.9
Male	24.2 ± 2.3	73.3 ± 8.8	177.0 ± 7.1

**Table 2:** Extracted features of the IMUs from neck and wrist, OFS and EMG sensor.

N°	Device	Feature	Description	Reference
1–4 5–8 9–12 13–16 17–20 21–24	IMU 1	GyroX GyroY GyroZ AccX AccY AccZ	Mean, standard deviation, RMS value and amplitude calculated per elbow repetition cycle from the IMU located at the neck. The amplitude is calculated as the maximum minus the minimum value from the IMU located at the neck.	[61,62]
25–28 29–32 33–36 37–40 41–44 45–48	IMU 2	GyroX GyroY GyroZ AccX AccY AccZ	Mean, standard deviation, RMS value and amplitude calculated per elbow repetition cycle from the IMU located at the neck. The amplitude is calculated as the maximum minus the minimum value from the IMU located at the wrist.	[61,62]
49–52		FIB_mean FIB_std FIB_RMS FIB_ROM	Mean, standard deviation, RMS value and Range of Motion. The ROM is calculated as the maximum minus the minimum value from the elbow joint position signal	[66,67]
	OFS			
53–56		FIB_tnorm FIB_MNF FIB_MDF FIB_IMNF	Normalized repetition duration, mean frequency, median frequency and instantaneous mean frequency of the elbow joint position signal	[68]
57–60		EMG_mean EMG_std EMG_RMS EMG_Amp	Mean, standard deviation, RMS value and amplitude. The amplitude is calculated as the maximum minus the minimum value from the biceps brachii EMG signal	[11,69]
	EMG Sensor			
61–63		EMG_MNF EMG_MDF EMG_IMNF	Mean frequency, median frequency and instantaneous mean frequency from the biceps brachii EMG signal	[64,65,70]

**Table 3:** Number of samples of each fatigue state in the dataset.

Fatigue State	Number of Samples
Low Fatigue	136 (36.6%)
Moderate Fatigue	104 (28.0%)
High Fatigue	132 (35.5%)

**Table 4:** Performance of the best five machine learning algorithms with the chosen hyperparameters. Light Gradient Boosting (LGBM), Random Forest (RF), Bagging (BC), Extra Trees (ET), and Decision Tree (DT).

Model	Hyperparameters	Accuracy	Precision	Recall	F-Score
**LGBM**	bagging_freq = 4 n_estimators = 100 min_child_samples = 8 num_leaves = 181	**96.8**	**96.8**	**96.8**	**96.8**
RF	n_estimators =1400 max_depth = 40 bootstrap = False	96.2	96.3	96.2	96.2
BC	max_features = 0.7 base_estimator_max_depth = 20 n_estimators = 10	96.2	96.3	96.2	96.2
ET	criterion = log_loss max_features = auto min_samples_leaf = 1 min_samples_split = 2	94.6	94.7	94.6	94.6
DT	criterion = gini min_samples_leaf =1 min_samples_split =8	93.0	93.0	93.0	92.9

**Table 5:** Performance of the LGBM classifier for fatigue estimation according to features importance and number of sensors.

Sensors	Features	Accuracy	Precision	Recall	F1-Score
EMG, IMUs, OFS	5	91.7	91.6	91.7	91.6
	**7**	**93.5**	**93.5**	**93.5**	**93.5**
	11	91.7	91.7	91.7	91.7
	16	95.9	95.9	95.9	95.9
	33	96.2	96.3	96.2	96.2
EMG	7	78.8	78.3	78.8	78.5
OFS	8	86.6	86.9	86.6	86.7
IMU1	5	86.6	86.2	86.6	86.2
IMU2	4	87.9	87.7	87.9	87.6
IMUs	10	92.2	92.1	92.2	92.1
IMUs, OFS	**13**	**95.4**	**95.4**	**95.4**	**95.4**

## Data Availability

Publicly available datasets were analyzed in this study. This data can be found here: https://figshare.com/projects/Biceps_Muscle_Fatigue_Dataset_three_states_/181144 (accessed on 13 October 2023).

## Abbreviations

The following abbreviations are used in this manuscript:

BC	Bagging Classifier
CWT	Continuous Wavelet Transform
DT	Decision Tree
EFB	Exclusive Feature Bundling
ET	Extra Trees
FN	False Negative
FP	False Positive
GOSS	Gradient-based One-side Sampling
GBDT	Gradient Boosting Decision Tree
HF	High Fatigue
IMU	Inertial Measurement Units
IMNF	Instantaneous Mean Frequency
k-NN	k-Nearest Neighbor
ML	Machine Learning
LOOCV	Leave-one-out Cross-validation
LGB	Light Gradient Boosting
LED	Light-emitting Diode
LDA	Linear Discriminant Analysis
LR	Logistic Regression
LF	Low Fatigue
MVC	Maximum Voluntary Contraction
MNF	Mean Frequency
MDF	Median Frequency
MOF	Moderate Fatigue
MF	Muscle Fatigue
MSD	Musculoskeletal Disorders
OFS	Optical Fiber Sensors
PCA	Principal Component Analysis
RF	Random Forest
ROM	Range of Motion
RPE	Rate of Perceived Exertion
RMS	Root Mean Square
SVM	Support Vector Machine
sEMG	Surface Electromyography
TP	True Positives

## Appendix A

The multidimensional fatigue questionnaire [49] contains 20 questions to assess 5 dimensions of fatigue: General, physical, reduced motivation, reduced activity, and mental. This is shown in Table A1.

**Table A1:** Multidimensional Fatigue Inventory (MFI) used in the experimental protocol.

Items	Fatigue Type
I feel fit	General
Physically, I feel only able to do a little.	Physical
I feel very active.	Reduced Activity
I feel like doing all sorts of nice things.	Reduced Motivation
I feel tired.	Reduced Activity
I think I do a lot in a day.	Mental
When I am doing something. I can keep my thoughts on it	Physical
Physically, I can take on a lot.	Reduced Motivation
I dread having to do things.	Reduced Activity
I think I do very little in a day.	Mental
I can concentrate well.	General
I am rested.	Mental
It takes a lot of effort to concentrate on things.	Physical
Physically I feel I am in a bad condition.	Reduced Motivation
I have a lot of plans.	General
I tire easily.	Reduced Activity
I get little done.	Reduced Motivation
I don’t like doing anything.	Mental
My thoughts easily wander.	Physical
Physically, I feel I am in an excellent condition	General
